# The effects of Valerian on sleep spindles in a model of neuropathic pain

**DOI:** 10.5935/1984-0063.20200110

**Published:** 2021

**Authors:** Ashkan Soltani, Farideh Bahrami, Zahra Bahari, Zeinab Shankayi, Mehdi Graily-Afra, Hedayat Sahraei

**Affiliations:** 1 Baqiyatallah University of Medical Sciences, Neuroscience Research Center - Tehran - Tehran -Iran.; 2 Baqiyatallah University of Medical Sciences, Physiology and Medical Physics, School of Medicine - Tehran - Tehran - Iran.

**Keywords:** Valerian, Sleep Spindle, Slow-Wave Sleep, REM, Anxiety, Sciatic Neuropathy

## Abstract

**Introduction:**

*Valeriana officinalis* is known to be one of the most famous herbal supplements for the treatment of anxiety and insomnia. Despite its widespread use in most countries all around the world, there is little scientific information and research on how this medication affects sleep patterns, and there are almost no studies on its effects on the characteristics of sleep spindles.

**Material and Methods:**

The present study was conducted to investigate the effects of Valerian extract (VAL) on sleep spindles and induced anxiety in chronic neuropathic pain model in rats. 24 male rats were divided into three groups: neuropathic group (n=9) in which the rats underwent chronic constriction injury (CCI), sham group (n=7) in which the sciatic nerves of the animals were exposed without any constriction and also fed with the vehicle, and the third group was under CCI condition and treated with Valerian (n=8). All the rats underwent electrode implant surgery so that we could record electroencephalogram and electromyography waves. In all the three groups, EEG and EMG recordings were recorded three times (150min each time). The initial recording was just prior to the CCI surgery and the rest were 3 and 6 days following CCI surgery. Moreover, cold allodynia and elevated plus maze tests were performed 3 and 6 days following the CCI surgery.

**Results:**

Valerian treatment could repair the allodynia induced by neuropathy. On the other hand, by Valerian treatment (400mg/kg) during neuropathy, the REM sleep, decreased and the non-REM sleep increased. Moreover, there was an increment in sleep spindle density and spindle frequency even in neuropathic condition.

**Discussion:**

This herbal supplement improves the quality of sleep in neuropathy conditions.

## INTRODUCTION

Various types of genus Valeriana (*Valerianaceae*) extracts are utilized in herbal medicine as mild sedatives and tranquilizers. Valerenic acid is the main constituent of *Valeriana officinalis*, most widely used in Europe and the USA. In living organisms, Valerenic acid or extracts from Valerian revealed tranquilizing and/or sedative effects^1-3^. Valerenic acid is the primary anxiolytic component whose effect could enhance with GABA^4^. Neurons expressing β3 containing GABA receptors are major cellular substrates for the anxiolytic action of Valerian^5^. *Valeriana officinalis* extract, functioning as an antioxidant agent, could be beneficial for reducing oxidative stress-associated insomnia complications^6^.

Sleep is a resting state of mind and body, characterized by reduced sensory reactivity to stimuli and altered unconsciousness, distinguished from wakefulness^7^. It plays an important role in sustaining a healthy lifestyle^8^. It also helps most of the systems in our body to restore normal functions. To keep the mood and cognitive performance, and specifically, contribute to the restoration of the function of the endocrine and immune system^8^, sleep is of great necessity. Sleep disorders adversely affect health, safety, and well-being. Patients with insomnia suffer from fatigue, lack of energy during the day, depression, weakened concentration, and reduced productivity at work.

Insomnia is a prevalent sleep disorder with three different types of sleep onset, sleep maintenance, and early morning awakening insomnia, which causes unrefreshing or restorative deficits^9^. Chronic insomnia could trigger severe disorders and irretrievable effects on physical and mental health. It has been implicated that this issue increases the risk of various health problems, including cardiovascular disease, diabetes, obesity, and Alzheimer's^10^. The treatment of insomnia has been performed employing hypnotics like benzodiazepine and zolpidem^11^. However, these hypnotic medications could result in several side effects such as daytime fatigue and cognitive impairments^11^.

Several kinds of food and nutrient intakes can affect the duration and quality of sleep. A recent study reported the association between dietary supplements and sleep duration^12^, suggesting that ingested nutrients play a pivotal role in better sleep. Moreover, several herbal extracts or resources have shown a positive effect on the improvement of insomnia^13^. The prevalence of sleep disturbance in patients with chronic pain ranges from 50% to 80%, and the severity of sleep disturbance is associated with pain intensity. As neuropathic pain and sleep disturbance have a bidirectional relationship, they should be treated concurrently. However, clinicians tend to focus on the pain, even though reducing sleep disturbance could lead to a decrease in pain intensity^14^. Neuropathic pain is associated with sleep disturbances reciprocally, and clinicians need to consider both aspects of treatment^15^.

In a sleeping human brain, spindle oscillations appear as brief (0.5-3 s) episodes of waxing-and-waning field potentials within a frequency range of approximately 9-15 H^16,17^. Spindles are a hallmark for light stages of non-REMs, during which they recur prominently once each 3-10 s in conjunction with other EEG rhythms between 0.5 and 16 Hz^16^. Spindle-generating neuronal circuits reside in the intra-thalamic network of the nucleus reticularis thalami (nRt) cells and thalamocortical (TC) cells^18^. Sleep spindles play role in multiple brain functions, including sleep quality, sensory gating, learning, and memory. Despite the significance of knowledge about the mechanisms underlying these neuronal rhythms, their function remains poorly understood. Newly identified molecular substrates of spindle oscillations, in combination with evolving technological progress, offer novel targets and tools to selectively manipulate spindles and dissect their role in sleep-dependent processes^18^. The differences in sleep spindle activity contribute to the differential vulnerability to sleep disturbances in the face of accelerating factors^19^. With lower spindle activity, particularly at the beginning of the night, larger increases are prospectively predicted in insomnia symptoms in response to stress^20^.

The changes in lifestyle and advancement of humans together with the development of technology and the urbanization of human societies are factors that could affect sleep patterns and associate with sleep disorders^21,22^. Moreover, various chemical drugs and sleeping pills that are commonly used for threatening sleep disorders are more problematic and have side effects^23,24^. Considering the history of traditional medicine in Iran, its popularity is high since herbal medicines are almost without side effects and Herein, taking the advancement of science and studies on sleep and the knowledge of its components; it is a good opportunity to discuss the effects of these herbal medicines on sleep. Valerian is employed as a hypnotic and anti-anxiety drug whose hypnotic properties have been largely studied. However, the physiological and particularly the electrophysiological effects of this plant are not clear, in particular in the field of its effects on sleep spindles and the stressor of pain.

In the present study, we clarified its effects on the characteristics of sleep spindles and anxiety. We also examined the effects of this drug on these factors in chronic pain conditions.

## MATERIAL AND METHODS

24 male Wistar rats, 2-3 months old, weight 180-200g divided into three groups, neuropathic group (n=9) in which the rats underwent chronic constriction injury (CCI), sham group (n=7) with exposure of sciatic nerve of animals without any constriction which fed with the vehicle, the other group was under CCI condition and treated with Valerian (n=8). All the rats underwent electrode implant surgery to record electroencephalogram and electromyography waves. In all three groups, EEG and EMG recordings were recorded three times (150min each time). The initial recording was just before the CCI surgery and the rest were 3 and 6 days following CCI surgery. Moreover, cold allodynia and elevated plus maze tests were performed 3 and 6 days following the CCI surgery. Animals were kept in cages with free access to foods and water in a room at 20-22ºC temperature and 12/12 light-dark cycle. All experiments were conducted as per the protocol approved by the University's Institutional Animal Ethics Committee.

### Treatment

Rats in the VAL+NRP (Valerian extract + Neuropathy) group were given orally the *Valeriana officinalis* extract daily for 3 weeks, one week was before EEG and EMG electrode placement surgery, and one week was during post-surgery recovery, and the third week was a week to record their sleep waves and neuropathy them by CCI surgery, and perform thermal allodynia and EPM tests. The dosage of the extract every day at 08:00 a.m. given to animals was 400mg/kg/day via the gavage method. The Valerian extract was given to the rats with a small amount of distilled water and the rats in the sham group were also given oral gavage with Valerian vehicle. Valerian solution was a hydroalcoholic extract of *Valeriana officinalis* with 0.824-0.994 specific gravity and 0000/200ml concentration, purchased from Zardband Ltd. (Tehran, Iran).

### Electrophysiological recording of sleep

A total amount of 450 minutes was recorded for each animal (150min in the base before neuropathy, 150min on day 3 after neuropathy surgery, and 150min on day 6 after neuropathy surgery). All sleep recordings were performed between 09:00 a.m. to 11:30 a.m. For the electrode implantation, the animals were anesthetized with ketamine (65mg/kg) (10%, Rotexmed, Germany) and xylazine (15mg/kg) (2%, Alfasan, the Netherlands) and fixed on a stereotaxic apparatus. Three stainless steel screw electrodes were implanted in the skull for EEG recording and the EMG was recorded from dorsal neck muscles via two stainless-steel wire electrodes. The EEG and EMG data were collected by the electrophysiology recording system (Ruby Mind, NY, US) in the 3,000Hz sampling rate. The signals were amplified and filtered (EEG 0-40Hz, EMG 1-400Hz)^20,25^.

### Operative procedures

VAL+NRP and NRP rats underwent CCI surgery for neuropathy, while sham group rats received a sham operation. For the CCI model, rats were anesthetized with ketamine (65mg/kg) and xylazine (15mg/kg), and four chromic catgut sutures spaced 1mm apart were tied loosely around the right sciatic nerve proximal to the trifurcation. Sham operations were also carried out under ketamine and xylazine anesthesia and involved exposure (but no manipulation) of the right sciatic nerve.

### Behavioral testing

#### Thermal allodynia (acetone test)

Behavioral testing to detect signs of cold allodynia was carried out in all rats; all three groups were present in this study on two occasions: days 3 and 6 after the CCI operation. Half an hour was given for adaptation and after that put the rat in the allodynia assessment box and then pulled acetone into the syringe and every 3 minutes with the speed by pressing the syringe towards the sole of the rat's foot, empty five times every 3 minutes and we take the hind paw withdrawal as a variable^26^.

#### Elevated plus maze

**Figure 1:**
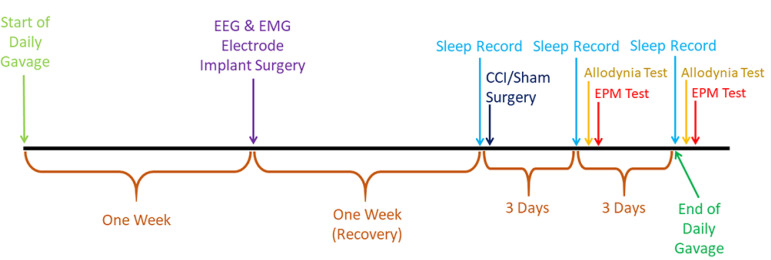
The time table of interventions performed All surgeries were performed in the morning. All sleep recordings were performed between 09:00 am to 11:30 am, after recording sleep, from noon, thermal allodynia, and elevated plus maze tests performed, respectively.

The elevated plus-maze consisted of two open and two closed arms, arranged oppositely to each other. The arms of EPM surfaces were 50cm long and 10cm wide and painted black. The walls of the closed arm of EPM also painted black, were 40cm in height. The maze was elevated to a height of 70cm above the floor. Animals were placed onto the center of the maze facing an open arm of EPM. During the five-minute test session, the animals were allowed to freely explore the maze. The number of the open and closed arm of EPM entries and the time each animal spent on the open and closed arm of EPM were recorded by the camera and later analyzed. An open arm of EPM entry was defined as all four paws on an open arm of the EPM^26-28^. The timetable of interventions performed in the study groups has been shown in Figure 1.

#### Sleep scoring and sleep spindle detection

The sleep stages were extracted semi-automatically using EEG and EMG recordings. The procedure for sleep scoring is as follows. First, three distinct and typical samples from EEG and EMG that were corresponded to wake, Slow-wave sleep, and REM were extracted by visual inspection. Each part was at least 30s. Then, a KNN classifier was developed in MATLAB (Math Works Inc., Natick, MA) to categorize every 5s of data into the three relevant classes based on available visually extracted samples. KNN classifies each part of recorded sleep data based on the similarity of EEG and EMG of that part to the visually identified parts^20^.

We calculated the EEG spectrogram along time and using spectrogram values, extracted the power of the extended sigma band (12-20Hz). To identify spindle events having the power in this band, a threshold was defined and a spindle was identified as the sigma power exceeded this predetermined threshold. We utilized a 12-20Hz frequency range to extract power as it led to the most accurate detection in our data and also fine-tuned the detection threshold value in such a way to boost conformity with visual identification of sleep spindles. We also add the criteria for spindles to last between 0.5s and 2s. The procedure was developed as a MATLAB script^20^.

### Statistical analyses

To compare the results of two different days in each group, a paired t-test was used for variables with normal distribution, and Wilcoxon non-parametric test was used for variables with non-normal distributed and the *p*-values less than 0.05 were considered as significant.

To compare the results of the groups on each day, the ANOVA parametric test was used for variables with normal distribution, and the Mann-Whitney U and Kruskal-Wallis non-parametric tests for variables with non-normal distributed and the *p*-values less than 0.05 were considered as significant.

For spindles, we computed amplitude in terms of root mean square (RMS), power of different frequency components within the spindle (Fourier analysis), as well as the mean frequency of spindles. Mean frequency is the weighted average of the diverse frequencies (Fourier components) in the EEG wave weighted by their corresponded power^20^. The computations were conducted in MATLAB. To compare results across the groups, an ANOVA was carried out with a 0.05 level of significance.

## RESULTS

### Cold allodynia

**Figure 3:**
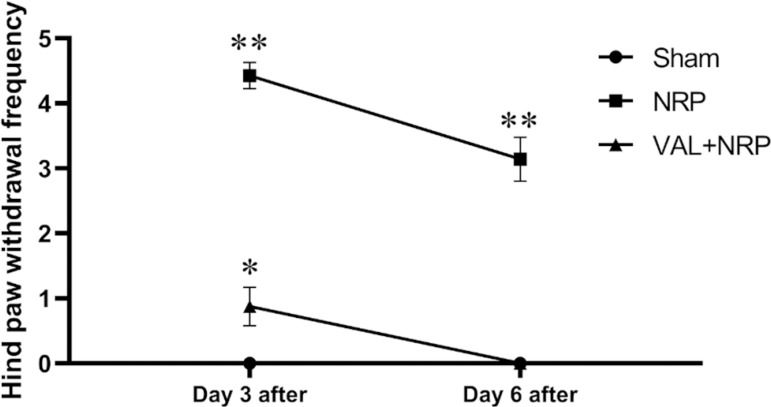
Reduction of cold allodynia with Valerian. The mean frequency of hind paw withdrawal in the VAL+NRP group was lower than that in the NRP group. The number of hind paw withdrawal was a measure of allodynia. The number of hind paw withdrawal in the sham group is zero on days 3 and 6 after sham surgery. In the VAL+NRP group, the amount of hind paw withdrawal is zero, day 6 after neuropathy. The results are shown in as the mean±SEM * = p < 0.05, ** = p < 0.01.

Since the rats of the sham group were not neuropathic, they were not sensitive to the cold allodynia test and the frequency of hind paw withdrawal in all the rats in the sham group was zero in all the five trials, on both days 3 and 6 after sham surgery. The average frequency of hind paw withdrawal, 6 days following the neuropathy in the VAL+NRP group was zero. The frequency of hind paw withdrawal in the NRP group was significantly higher in both 3 and 6 days following neuropathy compared to that in the VAL+NRP and the sham group (*p*<0.05). The frequency of hind paw withdrawal in the VAL+NRP group was significantly higher on day 3 after neuropathy than that in the sham group (Figure 3) (*p*<0.05).

### Anxiety-like behavior

**Figure 4:**
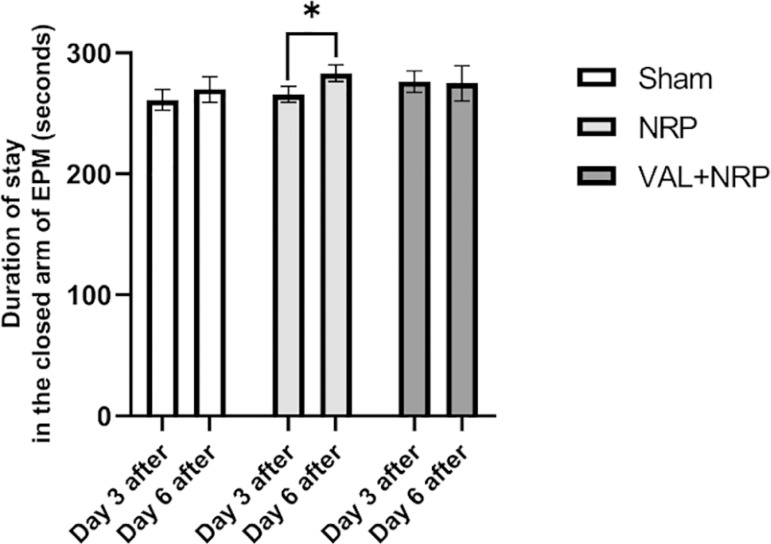
Assay of anxiogenic behavior in the presence of Valerian in neuropathic condition. The duration of the stay in the closed arm of EPM in the NRP group increased significantly on day 6 after neuropathy compared to day 3 after neuropathy. However, this increase was not seen in the VAL+NRP group. The results are shown as the mean±SEM, * = p < 0.05.

The duration of the stay in the closed arm of EPM in the NRP group increased significantly on day 6 compared to that on day 3 following neuropathy (*p*<0.05). The percentage of staying in the closed arm of EPM in the group receiving Valerian extract on days 3 and 6 after neuropathy did not differ significantly; also the percentage of staying in the closed arm of EPM in the sham group was not significantly different on days 3 and 6 after neuropathy (Figure 4).

### The stages of sleep

**Figure 5:**
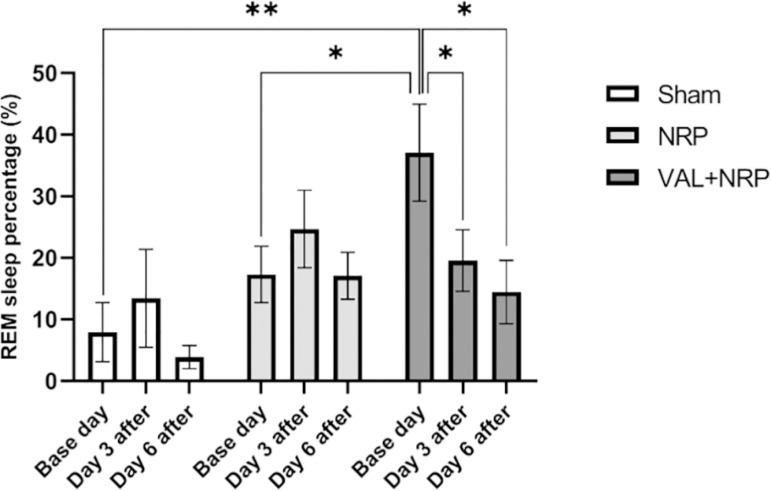
Assay of REM sleep. The percentage of REM sleep on the baseline day was significantly higher in the group receiving Valerian extract compared to that in the neuropathy and sham groups. In the group receiving Valerian extract, the percentagem of REM sleep decreased over time; on day 3 after neuropathy, it was less than that on the baseline day, and on day 6 after neuropathy, it was less than that on day 3 after neuropathy. The results are shown as the mean±SEM, * = p < 0.05, ** = p<0.01.

The percentage of REM sleep in the sham group did not differ significantly between the base, days 3 and 6 after neuropathy. The percentage of REM sleep in the VAL+NRP group declined, and these differences are significant (*p*<0.05). The percentage of REM sleep in the NRP group did not differ significantly between base and days 3 and 6. The REM sleep percentage on the base day was significantly different between the groups of sham (*p*<0.05), VAL+NRP, and NRP (*p*<0.05) (independent samples - Kruskal-Wallis test). The REM sleep percentage did not differ significantly between sham, VAL+NRP, and NRP groups on days 3 and 6 after neuropathy (Figure 5).

**Figure 6:**
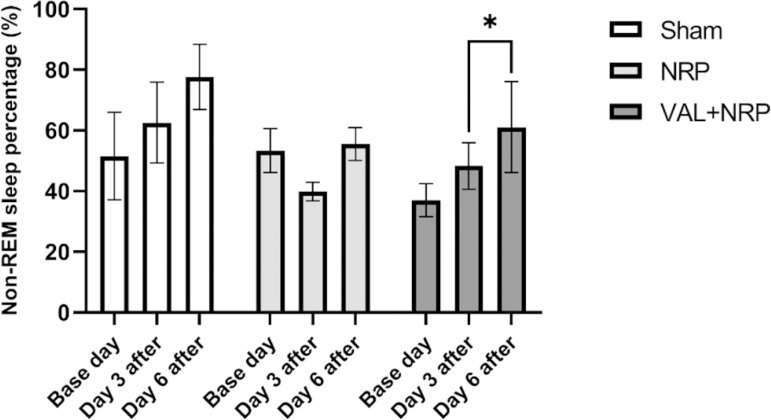
Valerian increases Non-REM sleep during neuropathic pain. The percentage of Non-REM sleep on day 6 after neuropathy was significantly higher than that in the group treated with Valerian on day 3 after neuropathy. The results are shown as the mean±SEM, * = p < 0.05.

The percentage of non-REM sleep in the VAL+NRP group on day 6 was more than day 3 after neuropathy, which is significant (*p*<0.05). Additionally, this percentage on day 3 following neuropathy was more than that on the base day, which was not significant. In the sham group, the percentage of non-REM sleep was higher on day 3 after neuropathy than that on the base day, and on day 6 it was more than that on day 3 after neuropathy, yet these differences were not significant. Comparing the groups, the percentage of non-REM sleep was not significantly different on days 3 and 6 after neuropathy (Figure 6).

### Sleep spindles

**Figure 7:**
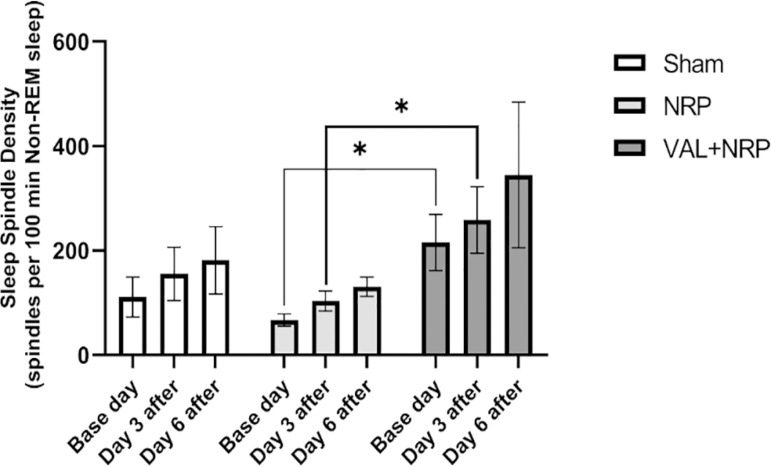
Valerian increases the density of sleep spindles. The density of sleep spindles in the VAL+NRP group on the base day and day 3 after neuropathy was significantly higher than that in the NRP group. The results are shown as the mean±SEM * = p < 0.05 Comparing the density of sleep spindles in the VAL+NRP group with the NRP group Mann-Whitney test.

Comparing the results between the groups, the density of sleep spindles in the VAL+NRP group was higher than that in the NRP group on the base day and day 3 after the neuropathy, which was significant (*p*<0.05). In all the three groups of sham, NRP, and VAL+NRP, the density of sleep spindles on day 6 after neuropathy was observed to be greater than that on day 3, and on day 3, it was higher than that on the base day. However, this difference was not significant (Figure 7).

**Figure 8:**
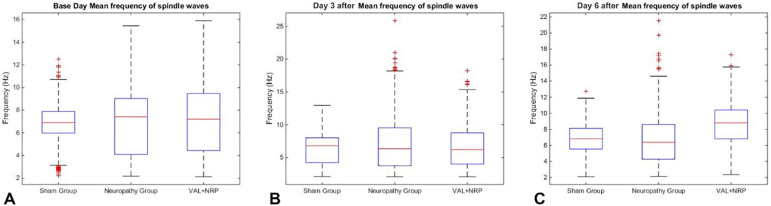
Valerian increases and changes the frequency of sleep spindles. Mean frequency of sleep spindles of all three groups: (A) Base day (B) Day 3 after CCI surgery (C) Day 6 after CCI surgery, the mean frequency of sleep spindles in the VAL+NRP group is significantly higher than both the sham and NRP groups and the average frequency of sleep spindles between the sham and NRP groups are the same (p<0.05).

The average frequency of sleep spindles in the sham group in 3 different days of sleep recording was not significantly different from each other. The average frequency of sleep spindles in the NRP group in the three different days of sleep recording was not significantly different from each other, but its trend decreased. In the VAL+NRP group, the average frequency of sleep spindles in each of the three days of sleep recording was significantly different from each other (*p*<0.05), and on day 3 after neuropathy, it was less compared to the base day, and on day 6 after neuropathy it was more than day 3 and base day. Moreover, it had an ascending trend the day after neuropathy, and the average frequency of sleep spindles in the group treated with Valerian increased over time. The average frequency of sleep spindles of the three groups on the base day was not significantly different from each other. The mean frequency of sleep spindles on day 6 after neuropathy in the group treated with Valerian was significantly higher than that in both the sham and NRP groups, and there was no significant difference in the average frequency of sleep spindles between the sham and NRP groups (Figure 8).

## DISCUSSION

### Reduction of cold allodynia with Valerian

The mean frequency of hind paw withdrawal in the VAL+NRP group was lower than that in the NRP group. The number of hind paw withdrawal was a measure of allodynia. We concluded that Valerian reduces the rate of neuropathic pain and cold allodynia in neuropathic rats. One of the important procedures employed by many researchers to study pain behavior is cold allodynia, which is an effective and easy method for detecting and evaluating pain^29^.

According to the study by Zare et al. (2018)^30^, the simultaneous use of Valerian root and turnip extracts has analgesic effects on both acute and chronic phases of the pain. The anti-inflammatory and analgesic properties of this herbal plant might be attributed to the presence of flavonoid compounds, prostaglandins, and cyclooxygenase enzymes in this plant. These properties could be also assigned to a decline in intracellular calcium.

### Neuropathy leads to increased anxiety

The duration of the stay in the closed arm of EPM in the NRP group increased. The longer was the duration of the stay in the closed arm of EPM, the more anxious the animal got. Increasing the duration of the stay in the closed arm of the EPM represented an increase in the anxiety levels and was a measure of the increased anxiety-associated behaviors^27,28^. Neuropathic pain in the CCI model increases anxiety-like behavior^31^.

### Assay of anxiogenic behavior in the presence of Valerian in neuropathic condition

Furthermore, the duration of the stay in the closed arm of EPM in the NRP group increased and this increment was not seen in the group treated with Valerian. Despite that, lack of meaningful increase in the duration of the stay in the closed arm of EPM in the NRP group in comparison with the sham group did not provide us with enough information to determine the anxiolytic effects of Valerian in a neuropathic state. However, certain studies suggested the use of Valerian as a treatment for anxiety and sleep disorders^32,33^.

### Effects of Valerian on sleep

The percentage of REM sleep on the baseline day was significantly higher in the group receiving Valerian extract compared to the neuropathic groups on the first day before neuropathic induction. The base day sleep of all the groups was before CCI surgery and neuropathy, and the rats in the group receiving Valerian extract received Valerian extract two weeks ahead of the baseline day. Therefore, this difference between the groups could be attributed to Valerian extract and according to this; we suggested that Valerian extract could increase REM sleep. In the group receiving Valerian extract, the percentage of REM sleep decreased over time; on day 3 after neuropathy, it was less than that on the baseline day, and on day 6 after neuropathy, it was less than that on day 3 after neuropathy. Accordingly, it seems that the neuropathic condition prevented the increment of REM sleep, which was induced with Valerian in the baseline day.

Moreover, although there was no increase in non-REM sleep with Valerian treatment before CCI injury, the percentage of non-REM sleep on day 6 after neuropathy was significantly higher than that in the group treated with Valerian on day 3 after neuropathy. Consequently, we could suggest that Valerian increases non-REM sleep during the neuropathic condition.

Choi et al. (2018)^34^ reported the Valerian/Cascade mixture decreased sleep latency whereas it increased the sleeping time by increasing non-REM and decreasing REM in normal and caffeine-induced arousal conditions in rodent models. The effects of the mixture on non-REM were recapitulated to the increase in the slow delta waves. However, in 2005, Shinomiya et al.^35^ reported that Valerian extract shortens the sleep latency, yet has no significant effects on the total time of wakefulness, non-REM sleep, or REM sleep.

### Valerian increases the density and frequency of sleep spindle

The present study mainly aimed to determine sleep spindles density and their characteristics. The density of sleep spindles in the VAL+NRP group on the base day and day 3 after neuropathy was significantly higher than that in the NRP group. Therefore, it may be suggested that Valerian increases the density of sleep spindles either in the absence or presence of neuropathy and the neuropathy condition could not change this effect. Moreover, we found that Valerian increases the frequency of sleep spindles since the average frequency of sleep spindles on day 6 after neuropathy in the group treated with Valerian was higher than that in the sham and NRP groups.

The Valerian/Cascade mixture could enhance not only sleep duration but also sleep quality^34^. Valerian extracts induced a significant increase in the delta activity during non-REM sleep, which may be useful as a herbal medicine with not only sleep-inducing effects, but also sleep quality enhancement effects^35^.

Zhu et al. (2012)^37^ reported that less sleep spindle activity, particularly at the beginning of the night, prospectively induces a more significant increase in insomnia. The individual differences observed in the sleep spindle activity contributed to the differential vulnerability to sleep disturbances in the face of precipitating factors, emphasizing the potential importance of therapeutic interventions aimed at enhancing sleep spindle activity to preserve sleep quality^19^. Low sleep quality and insomnia impair memory and learning^36^. The higher is the quality of sleep or the treatment of insomnia and sleep disorders, the better the person's performance, memory, and learning would be. Sleep disturbances could lead to neuroinflammation and impairment of memory and learning, which might play a role in the progression of cognitive decline in hospitalized patients^37^.

## CONCLUSION

Valerian could reduce the allodynia induced by neuropathy and may prevent anxiety-associated behaviors with neuropathy. This herbal medicine increases non-REM sleep during neuropathic pain, whereas it decreases REM sleep in this condition. Moreover, it could increase and change the density and frequency of sleep spindles even during neuropathy and may improve the quality of sleep during these conditions.

## Figures and Tables

**Figure 2:**
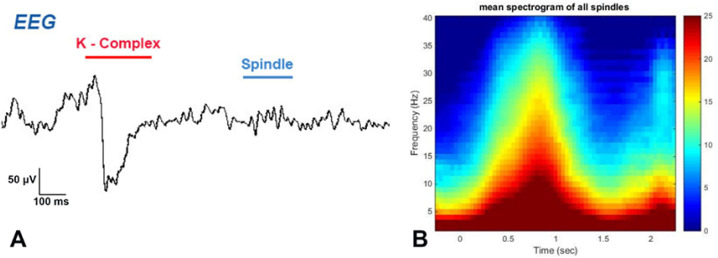
Sleep Spindle (A) Part of the EEG recording of one of the study rats, which represents the second stage of sleep, a sleep spindle, and k-complex is also shown in the image. (B) The mean spectrogram of all spindles, the shape of the candle flame indicates the average frequency of all sleep spindles in one of the sleep records from one of the study rats. And as it turns out, at lower frequencies, the power is higher, and the highest power is seen at frequencies below 15 Hz (12-13 Hz).

## ABBREVIATIONS

CCI: Chronic constriction injury

EEG: Electroencephalogram

EMG: Electromyography

EPM: Elevated plus maze

GABA: Gama aminobutyric acid

Non-REM: Non-rapid eye movement

NRP: Neuropathy

REM: Rapid eye movement

SEM: Standard error of mean

VAL: Valerian extract

VAL+NRP: Valerian extract + Neuropathy
